# Diet, fibers, and probiotics for irritable bowel syndrome

**DOI:** 10.25122/jml-2022-0028

**Published:** 2022-02

**Authors:** Adelina Nicoleta Galica, Reitano Galica, Dan Lucian Dumitrașcu

**Affiliations:** 1. 2^nd^ Department of Internal Medicine, Iuliu Hatieganu University of Medicine and Pharmacy, Cluj-Napoca, Romania; 2. Department of Nursing, Faculty of Natural and Human Sciences, Fan S. Noli University, Korçe, Albania; 3. Department of Obstetrics and Gynecology, Regional Hospital Korçe, Korçe, Albania

**Keywords:** irritable bowel syndrome, non-pharmacological therapy, probiotics, FODMAP, gut microbiota, IBS – irritable bowel syndrome, FODMAP – fermentable oligosaccharides, disaccharides, monosaccharides, and polyols, FGID – functional gastrointestinal disorders, NICE/mNICE – National Institute for Health and Care Excellence/modified diet, LFD – low FODMAP diet, IBS – D irritable bowel syndrome subtype with diarrhea, IBS – C irritable bowel syndrome subtype with constipation

## Abstract

Many aspects make irritable bowel syndrome (IBS) challenging for both patients and physicians. The unclear pathogenesis with many pathways to be explored, bothering symptoms that affect the quality of life, and many subtypes of the condition are only a few reasons that make IBS difficult to control and obtain satisfactory results. Treatment options start with general advice for lifestyle, continue with non-pharmaceutical treatments, and finally touch classic treatments. In this review, pharmaceutical treatment options are not accounted for. Consensus groups and meta-analyses have concluded guidelines that overall are the same, with variations in the strength of recommendations and some cultural and geographical particularities. Dietary interventions, probiotics, and fibers can be seen as non-pharmaceutical treatments that coexist in various protocols because of the relevant evidence regarding their efficacy in treating IBS symptoms.

## Introduction

Irritable bowel syndrome is a chronic condition accompanied by abdominal pain associated with defecation and alteration of the normal routine of feces elimination. Diagnosis is based on a group of clinical criteria and symptoms (currently named Rome IV criteria), and only in certain situations (when alarm signs are present or to help differential diagnosis) do clinicians use biochemical and imagistic investigations [1].

The pathogenesis of IBS is complex and partially understood, and a few theories were proposed to explain the symptoms: alteration of the intestinal microbiota, psychological conditions, disturbances of the gut-brain axis [2].

The new Rome IV criteria used in the most recent global study on functional gastrointestinal disorders (FGID) is more restrictive in diagnosing IBS, so the global prevalence now is about 4.6%, which is about half of what was known before with other Rome criteria. The studies conducted over time found that the prevalence is higher in women and those aged under 50, but the results are not 100% convergent [3–6]. Despite the changing numbers and data growing fast, the attempts to treat and manage IBS patients and their symptoms are still challenging because of the unknown pathogenesis and the multitude of factors that influence the functionality of the bowels. IBS affects the patients’ quality of life on various scales [7, 8], and this pushes clinicians to try to find the best-individualized alternative for the patient, but the task is not easy. In addition, the cost of healthcare, investigation, and treatment of IBS is significant [9]. The therapy of IBS has several levels, and the recommendation is a step-by-step approach of the patient, trying to implement first the non-pharmacological strategies. There are studies with good results in managing IBS symptoms with non-pharmacological therapy, including certain types of diet, fibers, and probiotics. Some countries have proposed guidelines for the treatment, and these are convergent. The experts’ opinion leads to common conclusions about what appears to be an effective treatment option.

### FODMAP and NICE

Food has a dual role in IBS; it is a trigger for symptoms but also a tool for therapy. One common practice in approaching IBS patients in different countries is to assess the lifestyle and offer dietary advice regarding certain foods or products that are known to affect the exacerbation of some symptoms [10]. Some patients can relate their symptoms to certain foods, so avoiding them can relieve some symptoms. The most common foods that some patients report to affect symptoms are coffee, alcohol, spicy and fatty food, but studies did not find any significant association with IBS [11]. Moderate physical activity and an appropriate hydration level are welcomed to complement dietary interventions [12, 13].

Few types of diet were tested on IBS patients: low FODMAP diet and mNICE (National Institute for Health and Care Excellence)-modified diet [14].

FODMAP consists of poorly absorbable short-chain fermentable carbohydrates (mono, di, and oligosaccharides) and polyols. These elements interact with gut microbiota, and the products resulting from fermentation influence the intestinal stem cells by inducing a low differentiation of endocrine cells [14]. The foods that contain those compounds are stone fruits, cereals, vegetables, milk-derivate products, and artificial sweeteners [15].

The FODMAPs are easily fermentable, and the gases resulting from this process (methane, hydrogen) lead to bloating because they are little absorbed by the gut [16]. Also, FODMAPs, through their osmotic effect, are causing accumulation of liquids in the gut and, together with the gases from the fermentation process, are causing colonic distention, which translates into IBS symptoms in patients with hyper sensibility [17]. Table 1 contains detailed examples of food included in low FODMAP and the size and portions that should be consumed.

**Table 1.** Detailed examples of food included in low FODMAP and the size and portions that should be consumed [2, 11, 17].

**Food category**	**Low Foodmap**	**Recommended size/day**
**Cereals (bread and pasta)**	Wheat-free grains, oat, rice, corn, quinoa	Up to 6 servings/day
**Vegetables**	Carrots, tomato, zucchini, cucumber, potato, bell pepper, broccoli, eggplant, green beans, spinach, parsnip, pumpkin, lettuce	3–5 servings/day
**Fruits**	Berries, grapes, lemon, oranges, pawpaw, raspberry, strawberry, cantaloupe, honeydew	2–3 servings/day
**Proteins**	Almond, hazelnuts, peanuts, pumpkin seeds	2 servings/day
**Milk and dairy**	Lactose-free milk and yoghurt, rice milk, almond milk, hard cheese, butter	2–3 servings/day

Two studies showed that a diet with FODMAPs affects bloating, abdominal pain, and other IBS symptoms in 70% of the patients [18, 19]. Comparing a low FODMAP diet (LFD) with traditional dietary advice shows low significance between the two types of diet regarding the response to therapy. In two other studies, LFD effectively managed pain and bloating more than a modified NICE diet or a typical local diet [16, 18].

The gut of IBS patients has a reduced density of endocrine cells. The abnormal endocrine expression from the gut causes, at least partially, gut dysmotility, visceral hypersensitivity, and abnormal secretion [14], which are the mechanisms involved in expressing IBS symptoms.

Implementing an LFD appears to normalize the density of the endocrine cells from the gut [20]. In addition, a low FODMAP diet was efficient in treating patients with IBS, and the duration was 3 to 6 weeks in most of the trials [21]. An LFD has three phases designed to test the results of elimination, assure that no harm is caused to the patient, and determine what food is causing the symptoms: the elimination, reintroduction, and personalized phase. This type of diet is useful in reducing bloating, abdominal pain, fecal frequency, and borborygmus [21] and can be offered to patients with IBS-D and bloating with satisfactory results (70%) in symptom improvement [22]. However, LFD has disadvantages both on a medical and personal level. One aspect is the objective restriction that comes with it: the malnutrition risk and the effect on the gut microbiota, which suffers alteration because of the lack of mono, di, and oligosaccharides. The other level is the personal aspect that implies that an LFD is expensive and difficult to implement in Western lifestyles [23]. It can also affect the patient’s social life and make it challenging to eat out and respect the dietary restrictions [24].

The modified NICE diet (small frequent meals, avoid trigger foods, and avoid excess alcohol and caffeine) is another option for IBS patients and appears to have the same effect as LFD in some countries. Nevertheless, it is easier to maintain and does not expose the patient to malnutrition as the LFD does if implemented for a long time [16, 18].

The mNICE diet has other recommendations regarding replacements and avoiding certain foods: a better option for those patients is to use spelt products instead of wheat products and psyllium husk as a source for fibers. Also, some vegetables like beans, onions, or cabbage should be left out of their diet, together with foods with high-fat content, soft drinks, and anything containing artificial sweeteners with names ending in “-ol”.

### Gluten-free and lactose-free diet

Gluten and lactose intolerance are two conditions with relevant prevalence in the general population, and each of them can be simultaneously present with IBS.

Several studies investigated the effect of a gluten-free diet in IBS patients, with no significant results. Even so, some benefits were observed in a few studies, but these cannot be attributed for sure to the gluten-free diet but mainly to the low FODMAP components [10]. The gluten-free diet is not recommended by a few consensus groups [21, 33] and a meta-analysis due to the unconvincing results on IBS symptoms and the burden on patients who barely preferred this diet over a balanced diet [25].

**Figure 1.**
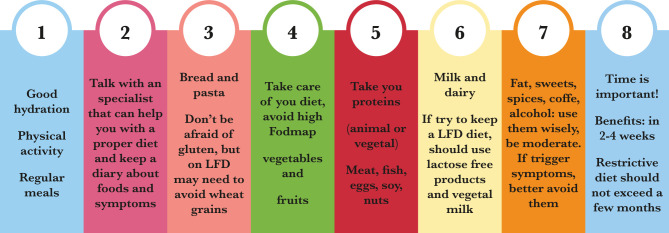
General advice and food categories recommended to an IBS patient.

Some of the IBS patients self-report as milk intolerant, but the studies conducted have shown a discrepancy between this statement and the assessment of the lactose absorption (with hydrogen breath test) [26–28]. As follows, a lactose-free diet is not recommended for IBS patients. The more detailed recommendations for IBS are listed in Figure 1.

### Fibers

Historically, fibers are known as an accessible, inexpensive, and efficient treatment for intestinal problems, with positive effects on patients’ health by decreasing the risk and mortality of cardiovascular diseases, obesity, diabetes, and colonic diseases [21].

Systematic reviews conducted on the efficacy of fiber shared a common conclusion: the key in using fiber for IBS is related to the fiber type, daily intake, and subtype of IBS. Edible fibers are defined as fibers and carbohydrates that are safe to be consumed but are not affected by the digestion process and are not absorbed by the intestine. Those fibers take part in fermentation processes in different proportions in the colon. They are provided by plants and parts of them are processed [21].

Insoluble fibers (corn, wheat bran) do not have benefits in treating symptoms of IBS and, in some cases, can have the opposite effect than intended by exacerbating symptoms such as bloating, constipation, and pain for patients with IBS-C [14, 29–31].

Soluble fibers are a distinct category, but it appears that solubility is not the only quality that brings benefits in relieving IBS symptoms [32]. From all soluble fibers categories, the ones with the best results in improving bloating, flatulence, abdominal distention, and increased fecal weight are soluble viscous low fermentable fibers (psyllium, ispaghula) [31, 33, 34]. Psyllium has a beneficial effect on symptoms in IBS-C and IBS-D [35]. The data are insufficient to suggest the best dose and the duration of the treatment, but the American Academy of Nutrition and Dietetics recommends a daily intake of 25 g for women and 38 g for men. This regimen is beneficial not only for IBS patients with any subtype but also for the well-being of any person [36].

Bulking polymers are an alternative for soluble fibers and are also recommended in IBS because of improving stool consistency by absorbing water [37]. Another rich source of fibers that appear to relieve IBS-C symptoms is linseeds. The recommended administration is up to 2 tablespoons/day of grounded linseeds, taken with fluids [38].

Prebiotics are fibers with the specific quality to nourish the beneficial bacteria from the gut and the probiotic strains administered to the patient [39].

Some dietary fibers that belong to the group of polysaccharides may act like prebiotics being implicated in the good functioning of the gut. They stimulate the development and health of bacterial species from the gut.

### Probiotics

Probiotics are live bacteria and yeasts that, when consumed, have some beneficial effects on the patient’s health, mainly over the digestive system. However, appropriate probiotic quantities are required to achieve the desired results [40].

IBS patients have a different gut microbiota than healthy individuals, which remains the base for manipulating intestinal microorganisms to improve IBS symptoms [41]. Healthy individuals have large variability in their intestinal bacterial composition, and many factors like genetics, diet, specific treatments, geographic position, surgery, smoking, and depression are responsible for this variability [42].

Four bacterial phyla are the main gut residents in healthy persons: Firmicutes, Bacteroidetes, Proteobacteria, and Actinobacteria [43]. The modifications in this ecosystem are called dysbiosis and can affect the number and biodiversity of gut microbiota. The attempts to map bacteria types affected in IBS had contradictory results, and the relation between a certain type of stain and specific symptoms is still to be further investigated. Studies showed that in IBS patients, there was a low population of *Lactobacillus sp.* and *Bifidobacterium sp.* [43]. Consistent with this, supplements containing these two species and Saccharomyces sp. have beneficial effects on IBS symptoms because of metabolism modulation and reducing low-grade inflammation [44].

Gut microbiota manipulation is a new hot topic regarding IBS treatment options because some studies have shown promising results. The downside is that there is a lack of strong data that can lead to clear headlines for the bacterial species to be used, if single or combined, the duration of treatment and specificity of symptoms that each strain can improve and what side effects can appear [45, 46]. Despite these, probiotics are seen as a strong recommendation for IBS [47, 21].

A review indicates that treatment with low or high doses of probiotics for a period shorter than 8 weeks significantly improves IBS symptoms and benefits quality of life (QoL). Furthermore, single-strain probiotics are more effective on symptoms than QoL [48]. The bacteria with the most benefits in IBS patients appear to be *Bifidobacterium infantis* [49], S. cerevisiae [50], and *Lactobacillus plantarum* [46].

Probiotics can improve stool frequency and consistency in IBS D and IBS C, but the effect on reducing abdominal pains, bloating, and flatulence is variable between studies. However, different species have been useful in treating those particular symptoms in IBS patients [51]. Besides restoring intestinal microbiota dysbiosis, probiotics can modulate GI motility, lower visceral hypersensibility, positively affect epithelial permeability and reduce immune mucosal activation [43]. Susceptible patients like those with weak immunocompetency can experience fungemia, bacteremia, and endocarditis) following the treatment with probiotics with S.boulardii, *Lactobacillus acidophilus*, L. casei, L. rhamnosus, and *Bifidobacterium*. Probiotics are overall effective, well-tolerated, safe, and low cost which recommends them to treat different types of IBS but for a limited time. However, all these statements still need further confirmation with stronger studies [52, 53].

## Conclusions

Dietary interventions, fibers, and probiotics are effective options for IBS treatment. A low FODMAP diet may not be easy to follow, but advantages appear to last in time. Probiotics are often the first choice for IBS, as they are not seen as medication. However, the modulation of gut microbiota may have more hidden benefits for the patient than just improving pain and other symptoms of IBS. More studies need to follow the path of the results obtained until now to better appreciate safety and effectiveness.

## Acknowledgments

### Conflict of interest

The authors declare no conflict of interest.

### Authorship

AG and DLD contributed to conceptualizing, AG and RG contributed to manuscript writing, and DLD edited and supervised the final form.
